# Correction: Temperature-sensitive foaming agent developed for smart foam drainage technology

**DOI:** 10.1039/d2ra90083a

**Published:** 2022-09-21

**Authors:** Wenfeng Jia, Chenggang Xian, Junwen Wu

**Affiliations:** State Key Laboratory of Petroleum Resources and Prospecting, China University of Petroleum Beijing 102249 China jiawf@cup.edu.cn xianchenggang@cup.edu.cn; Unconventional Oil and Gas Institute, China University of Petroleum Beijing 102249 PR China; Sinopec Research Institute of Petroleum Exploration and Development Beijing 102206 PR China wujunwen@iccas.ac.cn

## Abstract

Correction for ‘Temperature-sensitive foaming agent developed for smart foam drainage technology’ by Wenfeng Jia *et al.*, *RSC Adv.*, 2022, **12**, 23447–23453. https://doi.org/10.1039/D2RA04034D.

The authors regret that the email address of one of the authors (Chenggang Xian) was shown incorrectly in the original article. The corrected email address should be: xianchenggang@cup.edu.cn.

The Royal Society of Chemistry apologises for these errors and any consequent inconvenience to authors and readers.

## Supplementary Material

